# Nimbidiol protects from renal injury by alleviating redox imbalance in diabetic mice

**DOI:** 10.3389/fphar.2024.1369408

**Published:** 2024-05-21

**Authors:** Subir Kumar Juin, Sathnur Pushpakumar, Utpal Sen

**Affiliations:** ^1^ Department of Physiology, University of Louisville School of Medicine, Louisville, KY, United States; ^2^ Department of Microbiology and Immunology, University of Louisville School of Medicine, Louisville, KY, United States

**Keywords:** Nimbidiol, oxidative stress, ROS, blood pressure, KIM-1, diabetic kidney injury, NF-κB

## Abstract

**Introduction:**

Chronic hyperglycemia-induced oxidative stress plays a crucial role in the development of diabetic nephropathy (DN). Moreover, adverse extracellular matrix (ECM) accumulation elevates renal resistive index leading to progressive worsening of the pathology in DN. Nimbidiol is an alpha-glucosidase inhibitor, isolated from the medicinal plant, ‘neem’ (*Azadirachta indica*) and reported as a promising anti-diabetic compound. Previously, a myriad of studies demonstrated an anti-oxidative property of a broad-spectrum neem-extracts in various diseases including diabetes. Our recent study has shown that Nimbidiol protects diabetic mice from fibrotic renal dysfunction in part by mitigating adverse ECM accumulation. However, the precise mechanism remains poorly understood.

**Methods:**

The present study aimed to investigate whether Nimbidiol ameliorates renal injury by reducing oxidative stress in type-1 diabetes. To test the hypothesis, wild-type (C57BL/6J) and diabetic Akita (C57BL/6‐*Ins2^Akita^*/J) mice aged 10–14 weeks were used to treat with saline or Nimbidiol (400 μg kg^−1^ day^−1^) for 8 weeks.

**Results:**

Diabetic mice showed elevated blood pressure, increased renal resistive index, and decreased renal vasculature compared to wild-type control. In diabetic kidney, reactive oxygen species and the expression levels of 4HNE, p22phox, Nox4, and ROMO1 were increased while GSH: GSSG, and the expression levels of SOD-1, SOD-2, and catalase were decreased. Further, eNOS, ACE2, Sirt1 and IL-10 were found to be downregulated while iNOS and IL-17 were upregulated in diabetic kidney. The changes were accompanied by elevated expression of the renal injury markers viz., lipocalin-2 and KIM-1 in diabetic kidney. Moreover, an upregulation of p-NF-κB and a downregulation of IkBα were observed in diabetic kidney compared to the control. Nimbidiol ameliorated these pathological changes in diabetic mice.

**Conclusion:**

Altogether, the data of our study suggest that oxidative stress largely contributes to the diabetic renal injury, and Nimbidiol mitigates redox imbalance and thereby protects kidney in part by inhibiting NF-κB signaling pathway in type-1 diabetes.

## 1 Introduction

Diabetic nephropathy (DN) is the major microvascular complication of Diabetes Mellitus (DM) and has emerged as the principal cause of morbidity and mortality among diabetic patients. If DN remains untreated, hyperglycemia-induced renal disfunction progress to end-stage renal disease (ESRD), predominantly in type-1 diabetic (T1D) patients (Molitch et al., 2003; Boright et al., 2005; Tramonti and Kanwar, 2011; Grutzmacher et al., 2013; Kundu et al., 2015; Juin et al., 2021).

Oxidative stress has been shown to play a crucial role in vascular complications including DN and hypertension (Magenta et al., 2013). Further, previous reports also suggest that diabetes-induced oxidative stress regulates diabetic nephropathy (Zitka et al., 2012). Oxidative stress is primarily manifested by the excessive reactive oxygen species (ROS) production in the presence of inadequate or faulty antioxidant defence mechanism. It has been also demonstrated that hyperglycemia plays a causal role in the production of ROS (Koya et al., 2003; Gao et al., 2016). A plethora of studies have already shown the direct involvement of ROS in the kidney injury including DN (Koya et al., 2003; John et al., 2017).

In addition, hyperglycemia is known to induce vascular alterations leading to hypertension in T1D (Brownlee, 2005; Ceriello, 2006; de Boer et al., 2008). Although, hypertension is presumably associated with underlying nephropathy in type-2 diabetic patients due to concomitant ‘essential’ hypertension or renovascular complications, it is typically caused by the underlying DN in T1D patients (Molitch et al., 2004). It has been also demonstrated that hypertension-exacerbated glomerular dysfunction is mediated through oxidative stress in DN (Tomohiro et al., 2007).

Previous study suggested that adverse accumulation of extracellular matrix increases renal arterial restive index in diabetic kidney resulting to elevated blood pressure (John et al., 2017). Nevertheless, the co-existence of diabetes and hypertension synergistically induce oxidative stress and chronic inflammation, which in turn contribute to the pathogenesis of DN (Tomohiro et al., 2007; Lopes de Faria et al., 2011). The intricate relationship between oxidative stress and inflammation is a well-known fact. The frequent co-existence of oxidative stress and inflammation has been evidenced in various organs including the kidney (Vaziri and Rodriguez-Iturbe, 2006; Kern, 2007; Lopes de Faria et al., 2011). While the inflammatory cells instigate oxidative stress through the production of ROS, oxidative stress triggers inflammation via NF-κB-mediated upregulation of the pro-inflammatory molecules (Calcutt et al., 2009; Lopes de Faria et al., 2011).

A wide range of active constituents derived from the medicinal plant, *Azadirachta indica* (neem) have been implicated in the management of different diseases by regulating oxidative stress and inflammation (Alzohairy, 2016). Our recent study demonstrated that neem-derived diterpenoid Nimbidiol possesses anti-alpha-glucosidase activity that helps to ameliorate hyperglycemia thereby protecting diabetic mice from fibrotic renal dysfunction in part by mitigating adverse ECM accumulation (Juin et al., 2022). In the present study, we evaluated the contribution of oxidative stress in the diabetic renal injury and whether Nimbidiol protects kidney in T1D.

## 2 Materials and methods

### 2.1 Animals

Ten-fourteen-week-old male wild-type (WT) C57BL/6J (stock no. 000664) and type-1 diabetic (Akita) C57BL/6-*Ins2* ^*Akita*^/J (stock no. 003548) mice were bought from the Jackson Laboratory (Bar Harbor, ME). The mice were maintained in the University of Louisville’s animal facility and fed *ad libitum* with standard chow and water. The animal experiments were performed according to the approved protocols (Approval No. 20683, dated 2 December 2020) by the institutional animal care and use committee (IACUC) of the University of Louisville School of Medicine and conformed to the *Guide for the Care and Use of Laboratory Animals* published by the National Institutes of Health (NIH Publication, 2011), U.S.A. WT and Akita mice were either treated with saline or Nimbidiol (0.40 mg kg^−1^ d^−1^) for 8 weeks with a micro-osmotic pump. The dose of Nimbidiol was determined based on the optimized dose in our previous study (Juin et al., 2023). The four experimental groups were termed as ‘WT + Saline’, ‘WT + Nimbidiol’, ‘Akita + Saline’ and ‘Akita + Nimbidiol’. At the end of the experiment, 2X tribromoethanol (TBE) was used to euthanize the mice, and the samples were collected.

### 2.2 Chemicals

Dihydroethidium (cat. no.: D11347) was purchased from Thermo Fisher Scientific (Carlsbad, CA). The detection kit for GSH: GSSG (cat. no. ab138881) was procured from Abcam (Cambridge, MA). Assay kits for SOD (cat. no. 706002) and catalase (cat. no. 707002) activity were purchased from Cayman Chemicals (Ann Arbor, MI). Tween 20 (cat. no. M147) was from VWR Chemicals, LLC (Solon, OH), and polyvinylidene fluoride (cat. no. 1620177) membrane was from Bio-Rad (Hercules, CA). Non-fat dry milk powder (cat. no. M17200) and Bovine Serum Albumin (cat. no. A30075) were procured from Research Products International Corp. (Mt. Prospect, IL, USA). Agarose (cat. no. BP-160) was bought from Fisher Scientific (Fair Lawn, NJ). Optimal Cutting Temperature compound (cat. no. 23-730–571) was purchased from Fisher Healthcare, (Houston, TX). Nimbidiol (cat. no. SMB00209) was purchased from Sigma-Aldrich (St. Louis, MO).

### 2.3 Measurement of blood pressure

Blood pressure (BP) of the mice was measured every fortnight by the tail-cuff method using the ‘CODA™ Non-Invasive Blood Pressure System’ (Kent Scientific Corporation, Torrington, CT) as described previously (Majumder et al., 2022). Mice were trained with BP holder in a few 15 min sessions a week prior BP measurement. Mice were placed on an animal warming platform putting in the BP holder and acclimatised for 10 min before measuring the BP.

### 2.4 Renal ultrasonography

The mice were anesthetized with isoflurane and then placed on a warm (37.5°C) platform supinely. An ultrasound transmission gel (Other-Sonic; Pharmaceutial Innovations, Inc., Newark, NJ, USA) was applied on the depilated skin and ultrasound imaging was performed using a Vevo 2,100 system (VisualSonics, Toronto, ON, Canada, USA). The left kidney was scanned in the short axis by the transducer, MS550D (22–55 MHz). Peak systolic velocity (PSV) and end-diastolic velocity (EDV) (mm/sec) of the renal arterial blood flow was recorded in the Pulsed-Wave Doppler mode. Renal arterial resistive index (RI) was obtained by analysing the cine loops.

### 2.5 Renal angiography

Barium angiography was performed to evaluate renal vascular density as described previously (Pushpakumar et al., 2020). Barium sulfate (0.1 g/mL) was introduced into the kidney through the infrarenal aorta using a PE10 catheter (ID - 0.28 mm; Franklin Lake, NJ, USA). Renal angiogram was obtained using Kodak *In-Vivo* Imaging Systems FX Pro (Molecular Imaging System, Carestream Health Inc., Rochester, NY, USA). Density of the vessels was quantified using ‘VesSeg software tool’.

### 2.6 Measurement of reactive oxygen species (ROS)

Dihydroethidium (DHE) staining was used to measure a major ROS, i.e., superoxide (O2^•−^) in the kidney as described elsewhere (John et al., 2017). In brief, ice-cold acetone fixed kidney cryosections were incubated with freshly prepared 5 µM DHE solution for 15 min at room temperature in a humidified chamber avoiding exposure to light. The stained sections were imaged by an Olympus FluoView1000 confocal microscope (B&B Microscope, Pittsburgh, PA, USA) and the fluorescence intensity was determined using ‘ImageJ’ software.

### 2.7 Western blotting

Total protein was extracted from the kidney by RIPA buffer (Boston BioProducts, Worcester, MA, USA), containing phenylmethylsulfonyl fluoride and protease inhibitor cocktail (Sigma, St. Louis, MO, USA). The protein was quantified by Bradford assay. An equal amount protein was resolved by SDS-PAGE and Western blotting was performed following the standard protocol as described previously (Juin et al., 2021). GAPDH was used as a loading control for normalization of the relative expression of protein. A list of antibodies along with the sources, catalog numbers, and dilutions was provided in Table 1. ‘ImageJ’ software was used to quantify the protein bands by densitometric analysis.

**TABLE 1:** List of antibodies.

Antibody	Source	Catalog number	Dilution
4-HNE	Thermo Fisher Scientific (Carlsbad, CA)	MA5-27570	1:1,000
IL-17A	Thermo Fisher Scientific (Carlsbad, CA)	PA5-106856	1:1,000
ROMO1	Abcam (Cambridge, MA)	ab139353	1:1,000
IL-10	Abcam (Cambridge, MA)	ab189392	1:1,000
KIM-1	Abcam (Cambridge, MA)	ab47635	1:1,000
Lipocalin-2	R&D Systems, Inc. (Minneapolis, MN)	AF1857	1:4,000
p-NF-κB (p65)	Cell Signaling Technology (Danvers, MA)	3,033	1:1,000
IkBα	Cell Signaling Technology (Danvers, MA)	9,242	1:1,000
Sirt1	Cell Signaling Technology (Danvers, MA)	8,469	1:1,000
eNOS	BD Biosciences (San Jose, CA)	610,297	1:2,500
iNOS	BD Biosciences (San Jose, CA)	610,329	1:2000
GAPDH	Santa Cruz Biotechnology (Dallas, TX)	sc-365062	1:1,000
catalase	Santa Cruz Biotechnology (Dallas, TX)	sc-50508	1:1,000
SOD-1	Santa Cruz Biotechnology (Dallas, TX)	sc-101523	1:1,000
ACE2	Santa Cruz Biotechnology (Dallas, TX)	sc-390851	1:1,000
p22phox	Santa Cruz Biotechnology (Dallas, TX)	sc-20781	1:1,000
Nox4	Santa Cruz Biotechnology (Dallas, TX)	sc-55142	1:1,000
anti-rabbit IgG-HRP	Santa Cruz Biotechnology (Dallas, TX)	sc-2357	1:1,000
anti-mouse IgG-HRP	Santa Cruz Biotechnology (Dallas, TX)	sc-516102	1:1,000
SOD-2	MilliporeSigma (Burlington, MA)	06-984	1:1,000

### 2.8 Assessment of the reduced to oxidized glutathione ratio (GSH: GSSG)

An assay kit (refer to the ‘Chemicals and Antibodies’ section) was used to evaluate the ratio for the reduced to oxidized glutathione (GSH: GSSG) following the manufacturer’s instructions as mentioned elsewhere (Pushpakumar et al., 2020).

### 2.9 Determination of superoxide dismutase (SOD) and catalase activity

Total SOD and catalase activity was determined using Assay kits (refer to the ‘Chemicals’ section) according to the manufacturer’s protocol as described previously (Pushpakumar et al., 2020).

### 2.10 RNA isolation and semi-quantitative RT-PCR

Total RNA was extracted from the kidney using Trizol reagent (cat. no. 15596-026, Invitrogen, Carlsbad, CA, USA). cDNA was synthesized from 1 µg of isolated total RNA by reverse-transcription using an EasyScript cDNA Synthesis kit (cat. no. G234, MidSci, St. Louis, MO, USA) according to the manufacturer’s protocol. Reverse transcription-polymerase chain reaction (RT-PCR) was performed to amplify cDNA using the GoTaq Hot Start Green Master Mix (cat. no. M5122, Promega, Madison, WI, USA) as per manufacturer’s instructions. After PCR amplification, the product was subjected to electrophoresis on 1.5% agarose gel and a ChemiDoc XRS system (Bo-Rad, Hercules, CA) was used to visualize the bands. The band intensity was analysed by densitometry with ‘ImageJ’. A list of the primer sequences (Invitrogen, Carlsbad, CA, USA) are shown in Table 2.

**TABLE 2:** Primer sequences.

	Forward	Reverse
IL-10	5′AGA​TCT​CCG​AGA​TGC​CTT​CA3′	5′CCG​TGG​AGC​AGG​TGA​AGA​AT3′
IL-17	5′GCA​AGA​GAT​CCT​GGT​CCT​GAA​G3′	5′AGC​ATC​TTC​TCG​ACC​CTG​AAA​G3′
GAPDH	5′GTC​AAG​GCC​GAG​AAT​GGG​AA3′	5′GGC​CTC​ACC​CCA​TTT​GAT​GT3′

### 2.11 Statistical analysis

The experimental data are represented as mean ± standard deviation (SD) from 6 mice/group. Statistical significance was determined by the one-way or two-way analysis of variance (ANOVA) followed by Tukey’s *post hoc* test using GraphPad Prism 9 software. *p* < 0.05 was considered to be statistically significant.

## 3 Results

### 3.1 Nimbidiol treatment mitigated high blood pressure in diabetic mice

**FIGURE 1:**
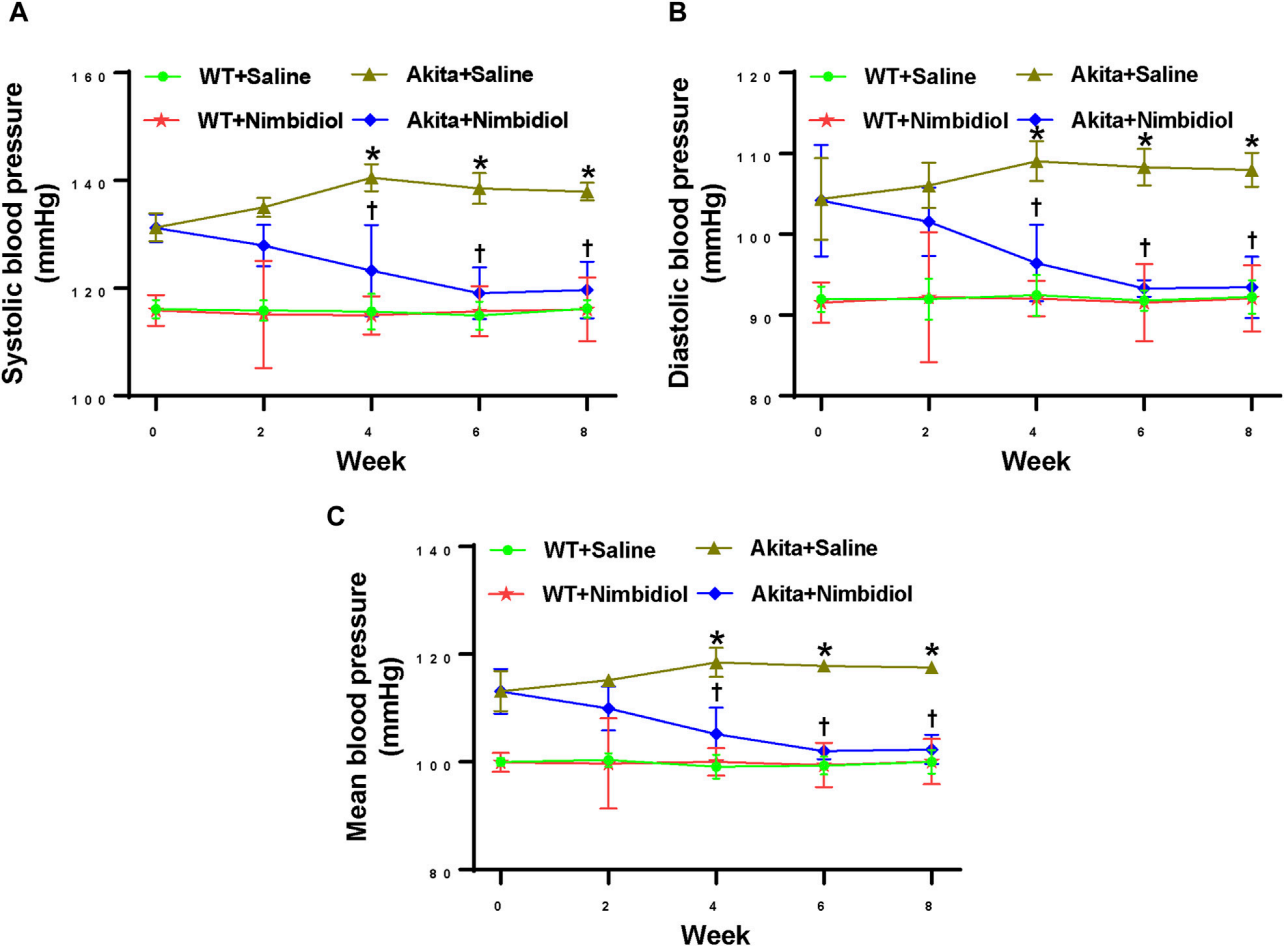
Nimbidiol treatment mitigated high blood pressure in diabetic mice. Time course changes of **(A)** systolic, **(B)** diastolic, and **(C)** mean arterial blood pressure was monitored by the tail-cuff method. Data are mean ± SD (n = 6/group). *p < 0.05 vs. WT + Saline, WT + Nimbidiol and Akita + Nimbidiol, ^†^ *p* < 0.05 vs. Akita + Saline.

At the beginning of the experiment (0 weeks), saline-treated diabetic Akita mice showed higher systolic, diastolic, and mean blood pressure (BP) compared the age-matched WT control (Figures 1A–C). BP in Akita mice continued to increase and reached to the peak at 4 week and thereafter remained constant (Figures 1A–C). Interestingly, Nimbidiol-treatment to Akita mice significantly reduced systolic, diastolic, and mean BP at 4 weeks onwards compared to that of saline-treated Akita mice (Figures 1A–C). The reduction of BP was maximum at 6 week of the Nimbidiol-treatment and thereafter it remained constant till the end of the experiment, i.e., 8 week (Figures 1A–C). BP was unchanged in the Nimbidiol-treated WT mice compared to the saline-treated WT control throughout the experiment (Figures 1A–C).

### 3.2 Nimbidiol attenuated elevated resistive index (RI) in the renal artery of diabetic mice

**FIGURE 2:**
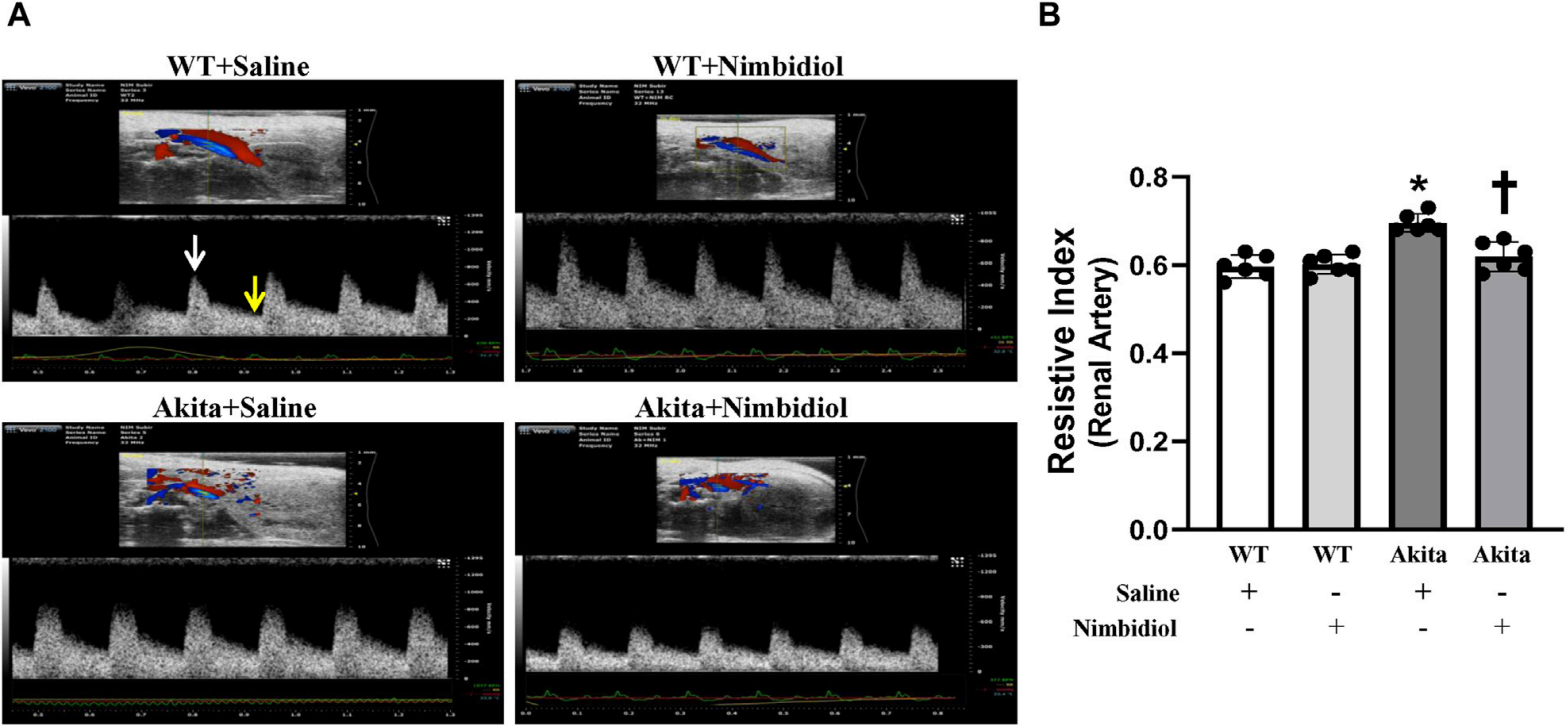
Nimbidiol attenuated elevated resistive index in the renal artery of diabetic mice. **(A)** Representative images from ultrasound of renal artery. Resistive index was calculated using the formula: (PSV-EDV)/PSV. PSV, peak systolic velocity (white arrow); EDV, end diastolic velocity (yellow arrow). **(B)** The bar graph shows mean resistive index. Data are mean ± SD (n = 6/group). *p < 0.05 vs. WT + Saline, WT + Nimbidiol and Akita + Nimbidiol, ^†^ *p* < 0.05 vs. Akita + Saline.

Renal arterial resistive index (RI) is the measure of vascular resistance that serves as a prognostic marker of renal outcome associated with diabetes and hypertension (Weber et al., 2017; Li et al., 2021). Therefore, we assessed renal arterial RI of the mice by ultrasonography and tested whether Nimbidiol influences vascular resistance. There was no significant change in RI between WT mice-treated with saline and Nimbidiol (Figures 2A, B). Akita mice exhibited significantly elevated renal arterial RI compared to that of WT control (Figures 2A, B). Nimbidiol-treatment to Akita mice significantly decreased renal arterial RI compared to the saline-treated Akita mice (Figures 2A, B).

### 3.3 Nimbidiol improved vascular density in the kidney of diabetic mice

**FIGURE 3:**
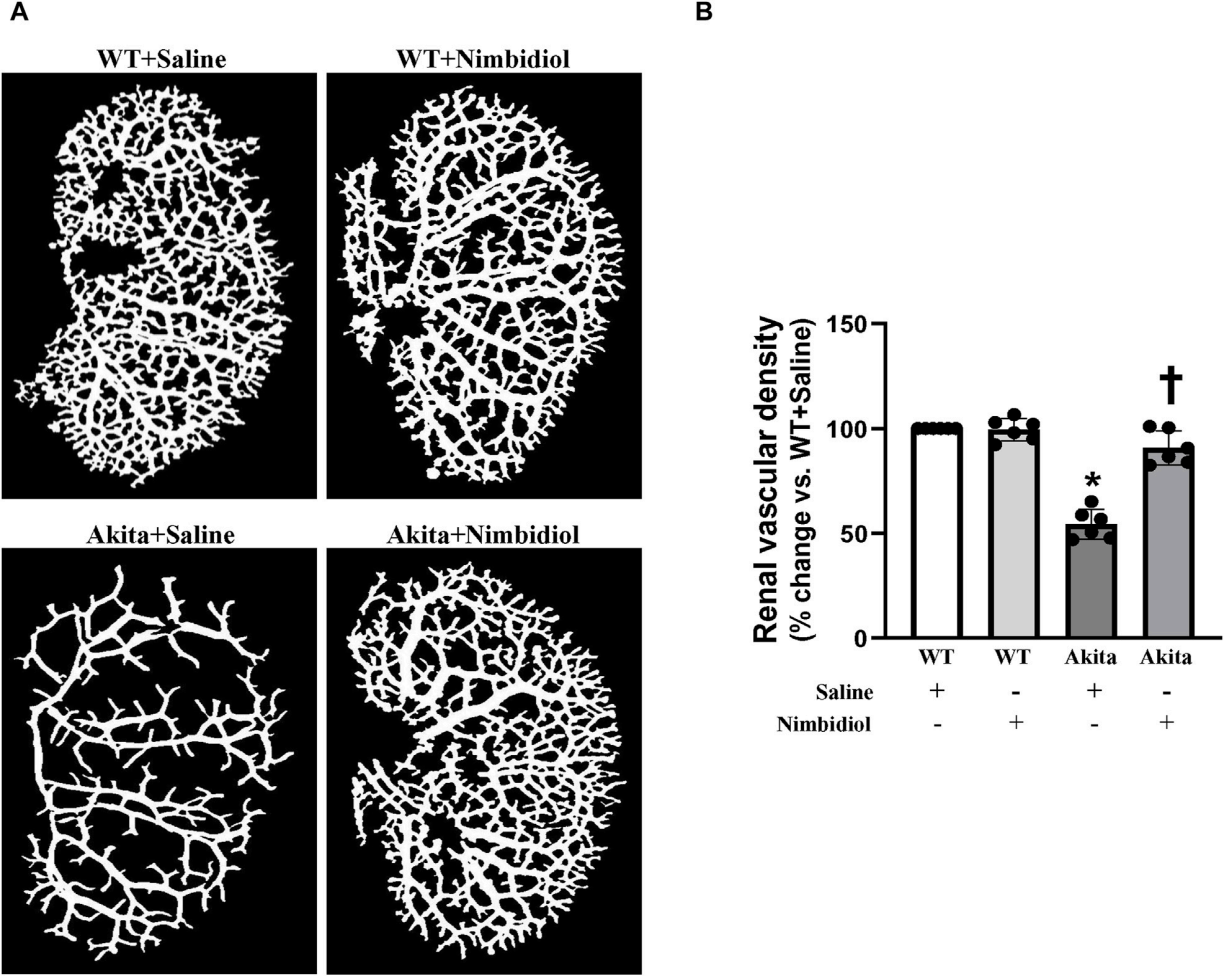
Nimbidiol treatment normalized vascular density in the diabetic kidney. Renal vascular architecture was captured by Carestream Molecular Imaging *In vivo* Multispectral system after infusion of 0.6 mL of barium sulfate (0.1 mg/mL) in the infrarenal aorta through a PE10 tube. **(A)** Vascular density was quantified utilizing Vessel Segmentation software. **(B)** Bar diagram represents the mean percentage change ±SD (n = 6). Values were obtained after background subtraction and plotted as percent change from WTY + saline group (100%). *p < 0.05 vs. WT + Saline, ^†^ *p* < 0.05 vs. Akita + Saline.

In order to evaluate renal vasculature, barium angiography was performed. Compared to the WT control, total renal vascular density of the Akita mice was significantly reduced (Figures 3A, B). Nimbidiol-treatment to Akita mice significantly increased vascular density compared to the Akita mice that received saline (Figures 3A, B). No significant change in total vasculature was observed between saline- and Nimbidiol-treated WT mice (Figures 3A, B).

### 3.4 Nimbidiol alleviated oxidative stress in the diabetic kidney

**FIGURE 4:**
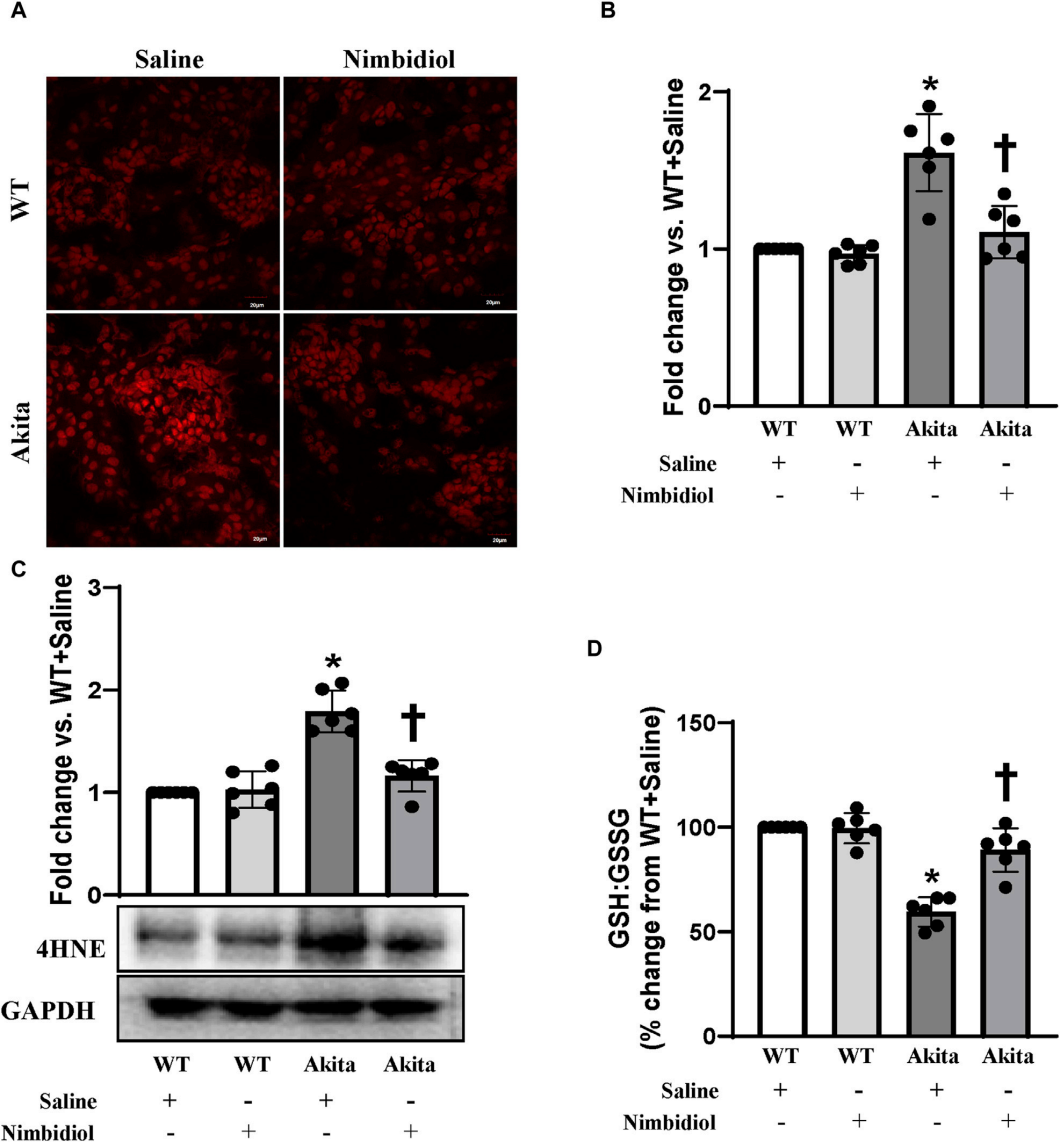
Nimbidiol alleviated oxidative stress in the diabetic kidney. **(A)** Dihydroethidium (DHE) staining was performed to evaluate superoxide (O2^•−^) in the kidney sections. Scale bar: 20 μm; magnification ×20 **(B)** The bar diagram represents the fold change in the fluorescence intensity for DHE staining from WT + Saline. **(C)** Western blot analyses showing protein expression of an α, β-unsaturated hydroxyalkenal product of lipid peroxidation, i.e., 4-Hydroxynonenal (4-HNE) in the kidney. The bar diagrams represent the fold change vs. WT + Saline. **(D)** Reduced to oxidized glutathione ratio (GSH: GSSG) was measured in the kidney and represented as the bar diagram showing the change in the percentage from WT + Saline. Data are mean ± SD (n = 6/group). *p < 0.05 vs. WT + Saline, WT + Nimbidiol and Akita + Nimbidiol, ^†^ *p* < 0.05 vs. Akita + Saline.

To assess reactive oxygen species (ROS) levels, dihydroethidium (DHE) staining was performed. The ROS production remained unchanged in the kidney of WT mice-treated saline and Nimbidiol (Figures 4A,B). Compared to the WT control, saline-treated Akita mice demonstrated an elevation in the ROS production as evidenced a significant increase in the fluorescence intensity in the glomerular and tubular regions of the Akita mice receiving saline (Figures 4A,B). It was interesting to note that Nimbidiol treatment to Akita mice significantly reduced the ROS production that is comparable to that of WT control (Figures 4A, B).

The lipid peroxidation product, 4-hydroxynonenal (4-HNE) is widely considered as a crucial oxidative stress marker that contributes to the hypertension and diabetic complications including DN (Calabrese et al., 2007; Yang et al., 2014; Csala et al., 2015; Yu et al., 2018; Dham et al., 2021). Therefore, we measured the expression of 4-HNE in the kidney. Our results showed that there was no significant difference in 4-HNE expression between WT mice treated with saline and Nimbidiol (Figure 4C). We observed a significant upregulation of 4-HNE in the kidney of saline-treated Akita mice, which was mitigated by Nimbidiol treatment (Figure 4C).

The ratio of reduced to oxidized glutathione (GSH: GSSG) serves as an important indicator of oxidative stress in the diabetic complications including DN (Zitka et al., 2012). To investigate whether elevated ROS production was associated with the imbalance in the ratio of the reduced to oxidized glutathione, we evaluated reduced and oxidized glutathione in the kidney of the experimental mice groups. Our results showed that there was no significant difference in the ratio of the reduced to oxidized glutathione in the kidney of saline- and Nimbidiol-treated WT mice (Figure 4D). However, the ratio was drastically decreased in the diabetic kidney compared to that of WT control (Figure 4D). Interestingly, Nimbidiol treatment ameliorated the adverse ratio in diabetic mice (Figure 4D).

### 3.5 Nimbidiol ameliorated the expression of p22phox, Nox4, ROMO1, SOD and catalase in the diabetic kidney

**FIGURE 5:**
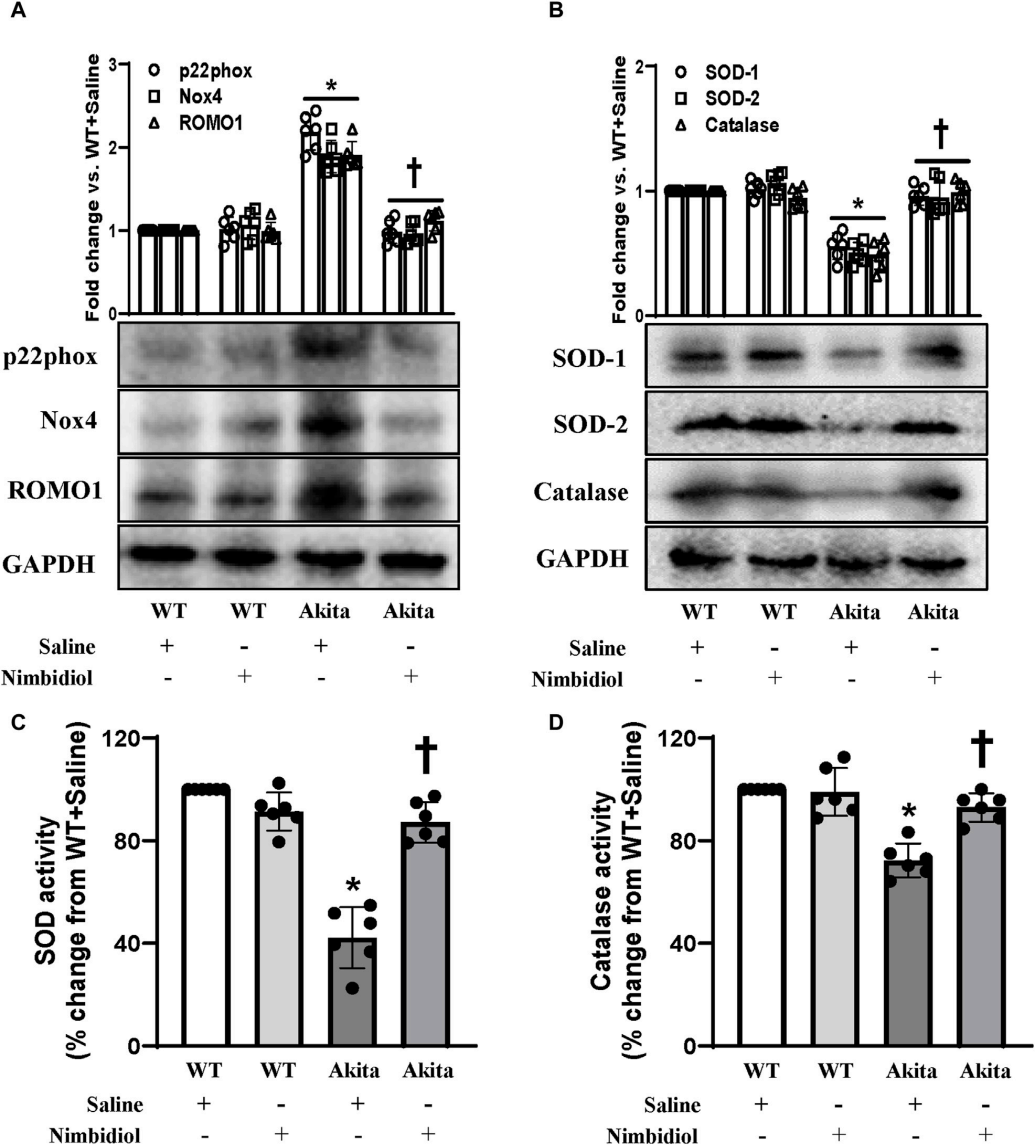
Nimbidiol ameliorated the expression of p22phox, Nox4, ROMO1, SOD and catalase in the diabetic kidney. Western blot analysis showing protein expression of **(A)** the primary producers of ROS such as p22phox and Nox4, and modulator of ROS, i.e., ROMO1 and **(B)** SOD-1, SOD-2 and catalase in the kidney. The bar diagrams represent the fold change vs. WT + Saline. **(C)** Total SOD activity and **(D)** catalase activity was measured and represented as bar diagrams showing the change in the percentage from WT + Saline. Data are mean ± SD (n = 6/group). *p < 0.05 vs. WT + Saline, WT + Nimbidiol and Akita + Nimbidiol, ^†^ *p* < 0.05 vs. Akita + Saline.

NADPH oxidase (Nox) system serves as the important source of ROS production (Pushpakumar et al., 2017). Nox4, the most abundant isoform of Nox present in the kidney, and another important Nox subunit p22phox have been reported to be involved in diabetes and hypertension (Etoh et al., 2003; Watanabe et al., 2013; Munoz et al., 2020). Therefore, the expression levels of Nox4 and p22phox were measured by Western blot analyses. Akita mice showed elevated expression of Nox4 and p22phox in the kidney compared to the WT control (Figure 5A). Nimbidiol treatment to Akita mice significantly reduced their expression levels, that were comparable to the WT mice (Figure 5A). Nox4 and p22phox expression in Nimbidiol-treated WT mice remained statistically unaltered compared to the WT mice receiving saline (Figure 5A). Further, we evaluated the expression of reactive oxygen species modulator 1 (ROMO1), an important regulator of the ROS production. Results revealed that ROMO1 was upregulated in the kidney of saline-treated Akita mice compared to that of WT control (Figure 5A). Interestingly, compared to the saline-treated Akita mice, ROMO1 expression was significantly reduced in Akita mice treated with Nimbidiol (Figure 5A). No significant difference in ROMO1 expression was observed between WT mice treated with saline and Nimbidiol (Figure 5A).

To understand whether elevated ROS generation was related to the imbalance of crucial antioxidative enzymes, we evaluated the expression of SOD-1, SOD-2, and catalase in the kidney. Western blot analyses showed a significant decrease in the renal expression of SOD-1, SOD-2, and catalase in diabetic mice compared to the WT control (Figure 5B). Nimbidiol-treatment to diabetic mice normalized their expression in the kidney (Figure 5B). However, Nimbidiol did not change the expression of these enzymes in the WT mice (Figure 5B). Then, we measured the activity of the total SOD and catalase in the kidney. Results revealed that there was no significant difference in the SOD and catalase activity in the kidney of the WT mice-treated with saline and Nimbidiol (Figures 5C, D). Compared to the WT control, on the other hand, diabetic mice showed a sharp decline in the SOD and catalase activity in the kidney (Figures 5C, D). Interestingly, Nimbidiol-treatment to diabetic mice normalized their activity, comparable to the WT control (Figures 5C, D).

### 3.6 Nimbidiol treatment normalized reduced eNOS and elevated iNOS expression levels in the diabetic kidney

**FIGURE 6:**
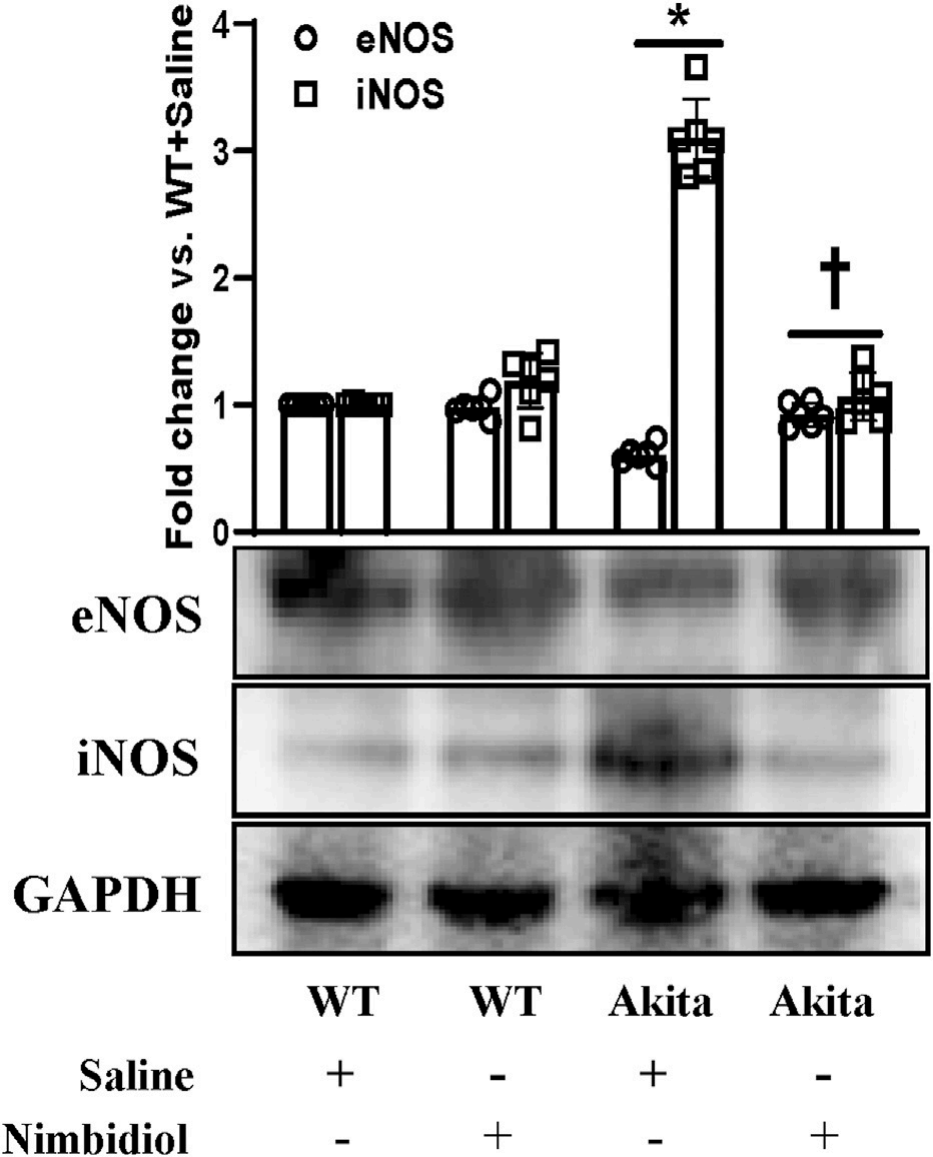
Nimbidiol treatment normalized reduced eNOS and elevated iNOS expression levels in the diabetic kidney. Western blot analyses showing protein expressions of eNOS and iNOS in kidney. The bar diagram represents the fold change vs. WT + Saline. Data are mean ± SD (n = 6/group). *p < 0.05 vs. WT + Saline, WT + Nimbidiol and Akita + Nimbidiol, ^†^ *p* < 0.05 vs. Akita + Saline.

The two important isoforms of nitric oxide synthases (NOSs) viz., Endothelial nitric oxide synthase (eNOS) and inducible nitric oxide synthase (iNOS) play crucial role in the regulation of oxidative stress, vascular function, blood pressure and DN (Trachtman et al., 2002; Kumar et al., 2005; Forstermann and Sessa, 2012; Dellamea et al., 2014; Pushpakumar et al., 2017; Juin et al., 2023). Therefore, we assessed the expression levels of eNOS and iNOS in the kidney. Akita mice showed a downregulation of eNOS and an upregulation of iNOS expression compared to the WT mice (Figure 6). Interestingly, Nimbidiol treatment to Akita mice normalized their expression levels that were comparable to the WT mice (Figure 6). Nimbidiol did not statistically alter the expression levels of eNOS and iNOS in WT mice compared to the saline-treated WT mice (Figure 6).

### 3.7 Nimbidiol ameliorated the altered expression of Sirt1 and ACE2 in the diabetic kidney

**FIGURE 7:**
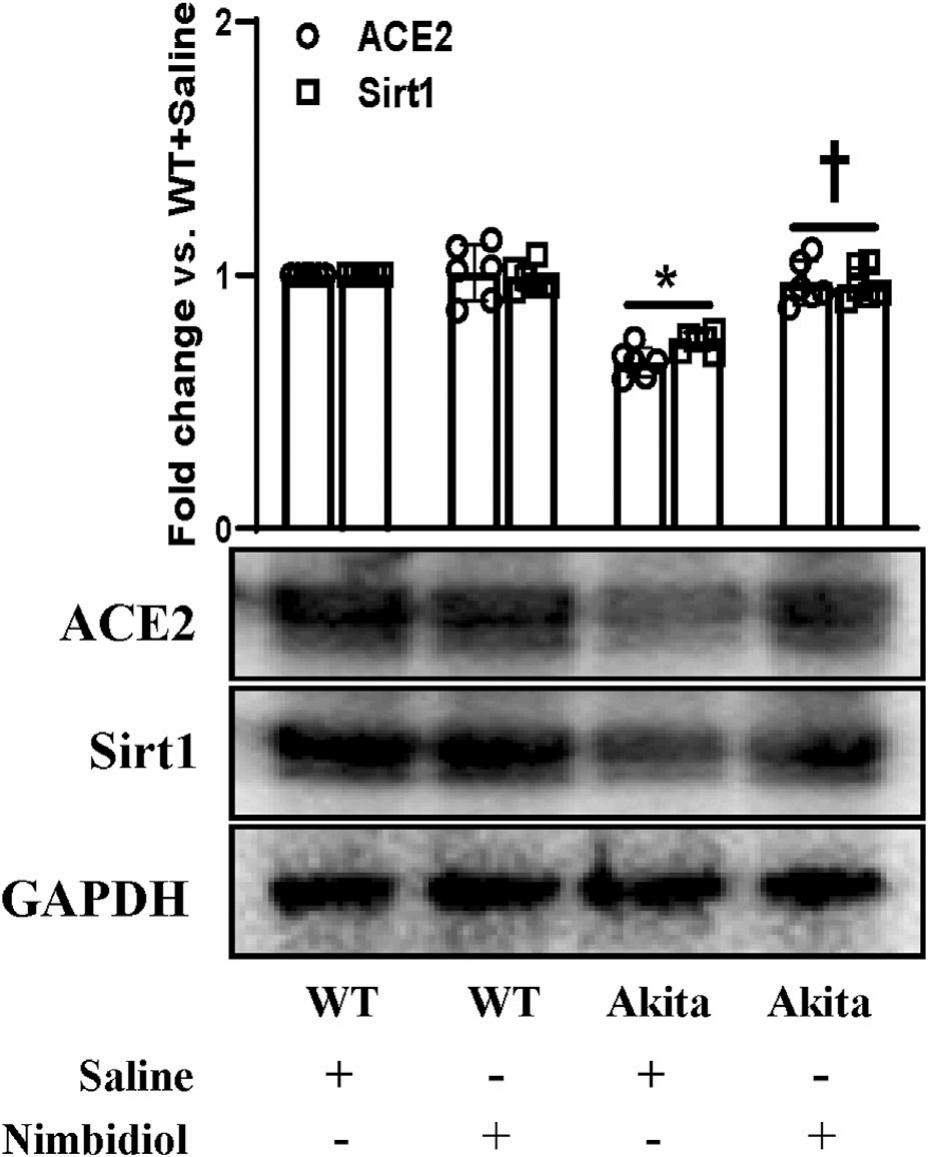
Nimbidiol ameliorated the altered expression of Sirt1 and ACE2 in the diabetic kidney. Western blot analyses showing protein expressions of ACE2 and Sirt1 in kidney. The bar diagram represents the fold change vs. WT + Saline. Data are mean ± SD (n = 6/group). *p < 0.05 vs. WT + Saline, WT + Nimbidiol and Akita + Nimbidiol, ^†^ *p* < 0.05 vs. Akita + Saline.

Oxidative stress induces progression and development of hypertension and nephropathy in diabetes (Shi et al., 2013). SIRT1 plays a pivotal role in the regulation of oxidative stress in hypertension and DN by regulating ACE2 expression (Yacoub et al., 2014). The results exhibited that there were no significant differences in the expression levels of SIRT1 and ACE2 between WT mice receiving saline and Nimbidiol (Figure 7). A distinct downregulation of the expression levels of SIRT1 and ACE2 was observed in Akita mice compared to the Winduce oxidative stress leading to the renal injury. Nimbidiol mitigatesT control (Figure 7). It was noteworthy that Nimbidiol treatment to Akita mice significantly increased the levels of SIRT1 and ACE2 expression, comparable to that of WT control (Figure 7).

### 3.8 Nimbidiol normalized the expression of pro-inflammatory cytokine, IL-17 and anti-inflammatory cytokine, IL-10 in the diabetic kidney

**FIGURE 8:**
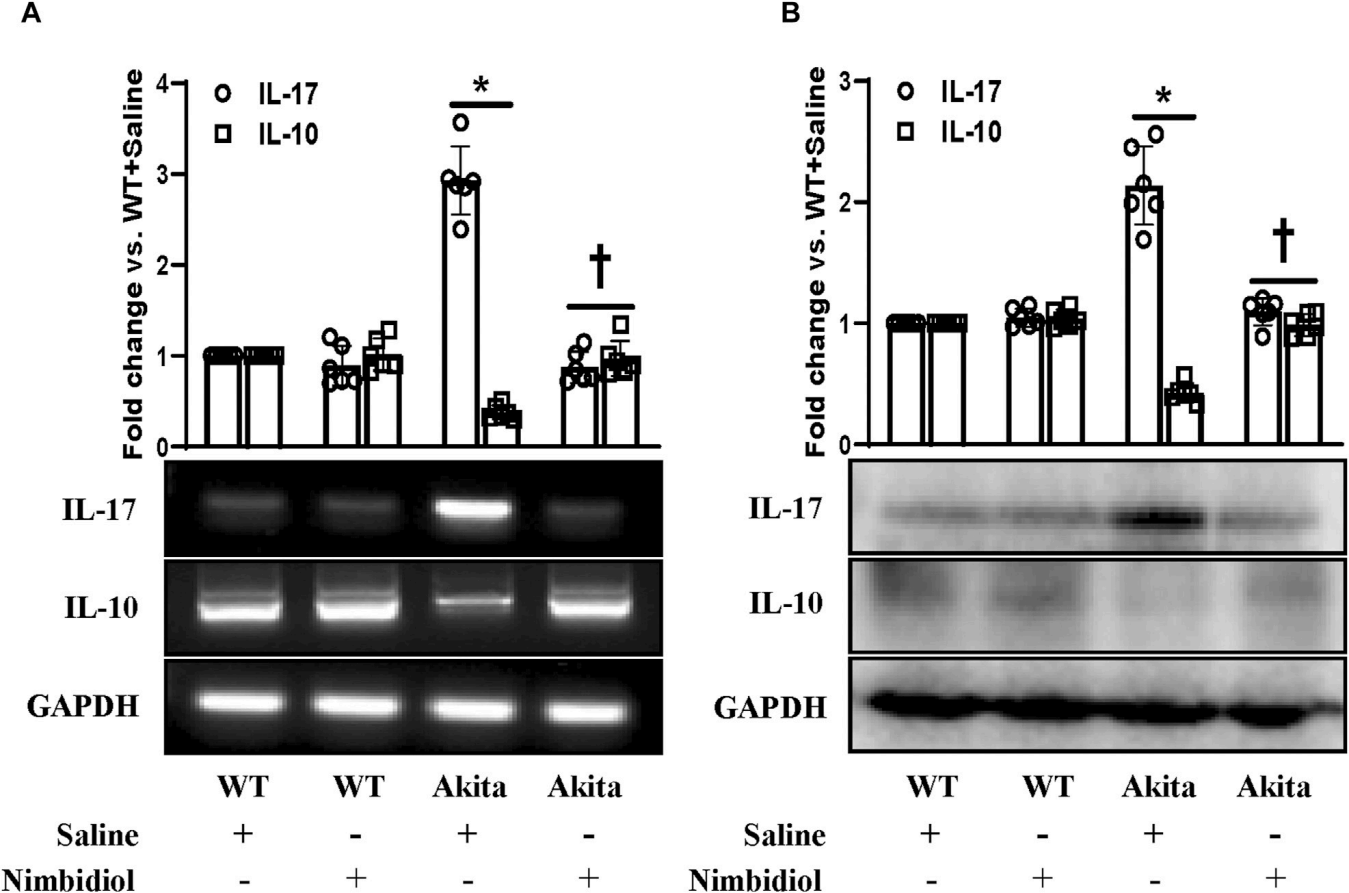
Nimbidiol normalized the expression of pro-inflammatory cytokine, IL-17 and anti-inflammatory cytokine, IL-10 in the diabetic kidney. **(A)** Semi-quantitative RT-PCR analyses showing gene expressions and **(B)** Western blot analyses showing protein expressions of IL-17 and IL-10 in kidney. The bar diagrams represent the fold change vs. WT + Saline. Data are mean ± SD (n = 6/group). *p < 0.05 vs. WT + Saline, WT + Nimbidiol and Akita + Nimbidiol, ^†^ *p* < 0.05 vs. Akita + Saline.

A plethora of evidence suggest the pathogenic role of proinflammatory cytokine IL17 and protective role of anti-inflammatory cytokine IL10 in diabetes and hypertension (Gunnett et al., 2002; Didion et al., 2009; Madhur et al., 2010; Weber et al., 2017; Qiu et al., 2021). To evaluate the mRNA and protein expression levels we performed semi-quantitative RT-PCR and Western blot analyses. Our results exhibited a distinct upregulation of IL-17 and a downregulation of IL-10 both at mRNA and protein levels in the kidney of Akita mice compared to the WT control (Figures 8A, B). In Akita mice receiving Nimbidiol, the expression levels of IL-17 and IL-10 were normalized (Figures 8A, B). The mRNA and protein expression levels of IL-17 and IL-10 remained unaltered in the WT mice treated with saline and Nimbidiol (Figures 8A, B).

### 3.9 Nimbidiol protected diabetic mice from kidney injury

**FIGURE 9:**
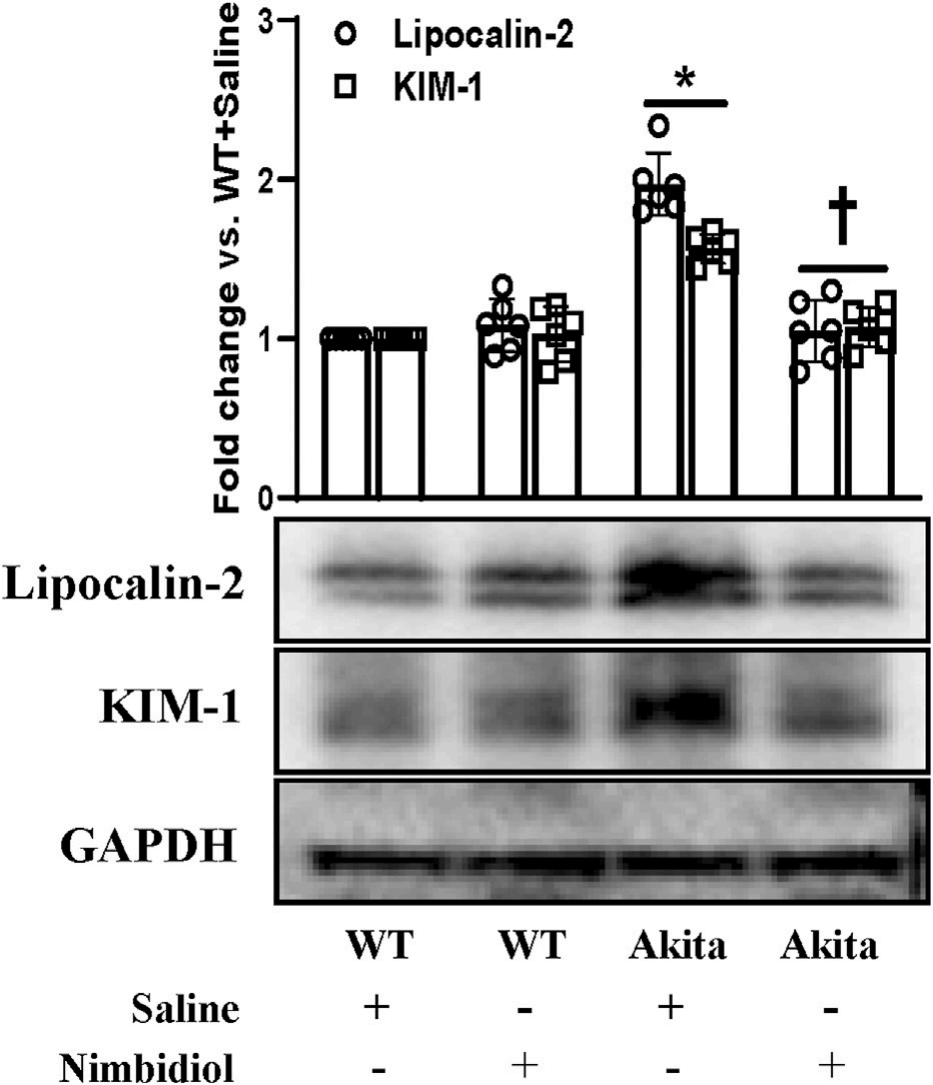
Nimbidiol treatment mitigated the expression of lipocalin-2 and KIM-1 in the diabetic kidney. Western blot analyses showing protein expressions of lipocalin-2 and KIM-1 in kidney. The bar diagram represents the fold change vs. WT + Saline. Data are mean ± SD (n = 6/group). *p < 0.05 vs. WT + Saline, WT + Nimbidiol and Akita + Nimbidiol, ^†^ *p* < 0.05 vs. Akita + Saline.

Kidney injury molecule-1 (KIM-1) present in the proximal tubules of the kidney is largely recognized as an important biomarker of the progressive renal damage in diabetes (Papu John et al., 2019; Majumder et al., 2022). Further, Lipocalin-2 (LCN-2) also serves as a crucial prognostic marker of renal injury in diabetes and hypertension (Johnson et al., 2022; Majumder et al., 2022). Therefore, we investigated whether diabetic conditions induced kidney damage and Nimbidiol treatment ameliorated renal injury in Akita mice. The Western blot analyses revealed that there was no significant change in the expression levels of KIM1-1 and LCN-2 between WT mice treated with saline and Nimbidiol (Figure 9). We observed that KIM1-1 and LCN-2 were substantially elevated in the kidney of the Akita mice compared to the WT control (Figure 9). Of note, Nimbidiol treatment to Akita mice significantly reduced the levels of KIM1-1 and LCN-2 that were comparable to the WT control (Figure 9).

### 3.10 Nimbidiol inhibited NF-κB signaling in the diabetic kidney

**FIGURE 10:**
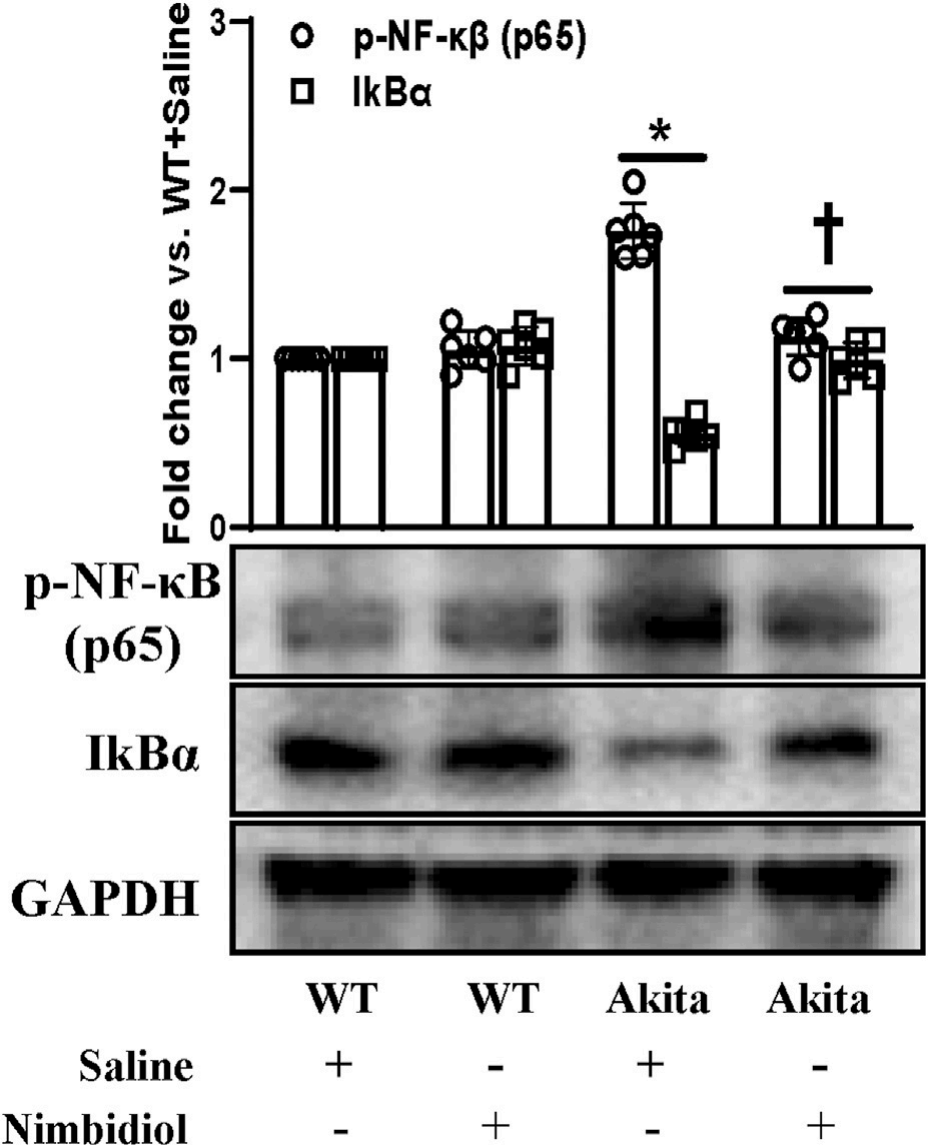
Nimbidiol inhibited NF-κB signaling in the diabetic kidney. Western blot analyses showing protein expressions of p-NF-κB (p65) and IkBα in kidney. The bar diagram represents the fold change vs. WT + Saline. Data are mean ± SD (n = 6/group). *p < 0.05 vs. WT + Saline, WT + Nimbidiol and Akita + Nimbidiol, ^†^ *p* < 0.05 vs. Akita + Saline.

NF-κB signaling plays a critical role in the development of different kindney diseases including DN by regulating macrophage polarization and gene expressions of various inflammatory cytokines and chemokines (Baker et al., 2011). Therefore, we measured the expressions of phosphorylated-NF-κB (p65) [p-NF-κB (p65)] and IkBα in the kidney by Western blot analysis. p-NF-κB was found to be significantly upregulated and IkBα was significantly downregulated in the diabetic kidney compared to the WT control (Figure 10). Nimbidiol treatment normalized the expression levels of p-NF-κB and IkBα in Akita mice (Figure 10). However, there were no significant differences in the protein levels of p-NF-κB and IkBα between saline- and Nimbidiol-treated WT mice (Figure 10).

## 4 Discussion

Diabetic nephropathy (DN) is the principal microvascular complication of diabetes and is the leading cause of end-stage renal disease (ESRD). DN has been a major threat to the global population in the recent years. The pathogenesis of DN is multifactorial. The concomitance of elevated blood pressure with the increased albuminuria and reduced glomerular filtration rate (GFR) in DN ultimately progresses to ESRD (Cooper, 1998).

In diabetes Mellitus (DM), the impact of hypertension on exponentially poor vascular outcomes have been evidenced including DN (Giunti et al., 2006). As arterial hypertension remains the major risk factor for the development of DN, alleviation of elevated blood pressure has been crucial for the control and management of DN in type-1 diabetes (T1D) (Giunti et al., 2006; Thomas and Atkins, 2006; Wong et al., 2007). Therefore, haemodynamic factors such as systemic and glomerular hypertension, and the vasoactive hormone, angiotensin II have become the major therapeutic target for DN (Cooper, 1998; Wong et al., 2007). In consistent with the earlier observations, our present study demonstrated a rise in blood pressure in diabetic Akita mice (Gurley et al., 2006; Oudit et al., 2010). Further, elevated BP was found to be associated with increased renal resistive index (RI) and reduced vasculature in Akita mice, corroborating the previous report (John et al., 2017). Notably, Nimbidiol mitigated the elevation in blood pressure, ameliorated RI and improved renal vasculature in Akita mice.

The occurrence of hypertension remains to be more prevalent among diabetic patients compared to the non-diabetic subjects (National High Blood Pressure Education, 1994). The synergistic effect of hyperglycemia and hypertension augments the pathogenesis of DN (Stratton et al., 2006). A close correlation of increased blood pressure and impaired renal function was evidenced with the commencement of DN in T1D (Jensen et al., 1987). However, the interplay by which hyperglycemia and hypertension aggravates diabetic nephropathy is not fully understood. The present study elucidates that oxidative stress mediates the synergistic detrimental consequences of hyperglycemia and elevated blood pressure on kidney injury in T1D mice.

Previous reports suggested that oxidative stress and inflammation are greatly involved in the development of DN. In this context, it is noteworthy that hyperglycemia-exacerbated excess superoxide anion (O2^•−^) induces kidney injury in DN (Koya et al., 2003; Thallas-Bonke et al., 2008; Coughlan et al., 2009). Our study also showed elevated ROS production in diabetic mice. Moreover, hyperglycemia-induced excess production of renal superoxide, due to an imbalance between NADPH oxidase (Nox), the superoxide-producing enzyme and superoxide dismutase (SOD), the superoxide-scavenging enzyme, instigates oxidative stress leading to the progression and maintenance of DN (Fujita et al., 2012). Further, the crucial contribution of mitochondrial Nox system especially, Nox4 has been suggested to trigger oxidative stress in hyperglycemia including in T1DN (Brownlee, 2005; Gorin et al., 2005; Susztak et al., 2006; John et al., 2017). In agreement with these previous findings, our study also demonstrated an elevation in ROS, 4HNE, Nox4, p22phox, and ROMO1, and a decrease in SOD and catalase activity in the kidney of Akita mice, suggesting elevated oxidative stress in T1DN. Further, a decrease in the ratio of the reduced to oxidized glutathione (GSH:GSSG) in Akita mice corroborating the previous observations in DN (Zitka et al., 2012).

Although Nox remains the primary source for the cytosolic ROS production, activity of nitric oxide synthases (NOSs) plays a crucial role in the regulation of vascular endothelial dysfunction, ROS generation, and upregulation of the pro-inflammatory mediators, contributing to the pathogenesis of hypertension and DN (Thallas-Bonke et al., 2008; Balakumar et al., 2009). We observed an upregulation of iNOS and a downregulation of eNOS at the protein level in the kidney of diabetic mice, supporting the previous findings, which elucidates that reduced eNOS and elevated iNOS instigate renopathy in diabetes and hypertension (Kumar et al., 2005; Zhao et al., 2006; Balakumar et al., 2009; Wang et al., 2011). However, differential expression of eNOS and iNOS was explained as the result of the time course of diabetes induced in different experimental models (Onozato et al., 2002).

Previous studies have provided the evidence of important role of ACE2 in the pathogenesis of hypertension and DN (Castro-Chaves et al., 2010; Oudit et al., 2010). A reduction in ACE2 was shown to be associated with diabetic nephropathy and absence of ACE2 has been shown to worsen the pathogenicity of DN (Tikellis et al., 2003; Shiota et al., 2010). Our study revealed a downregulation of ACE2 in T1D Akita mice which is consistent with the previous observation in different experimental diabetic mice including diabetic Akita mice (Tikellis et al., 2003; Gurley et al., 2006; Wysocki et al., 2006; Wong et al., 2007; Oudit et al., 2010). Nimbidiol-treatment was effective to elevate the level of ACE2, suggesting that elevation of ACE2 by the therapeutic intervention of Nimbidiol could be novel approach in the normalization of elevated BP and amelioration of diabetic renal injury. Sirtuin 1 (Sirt1) is an important member of the conserved family of the nicotinamide adenine dinucleotide (NAD^+^)-dependent deacetylases that controls hyperglycemia, oxidative stress, inflammation and fibrosis by regulating transcriptional activities of a wide range of enzymes including ACE2, eNOS and NF-κB p65 subunit in DN (Hallows et al., 2012; Price et al., 2012; Yacoub et al., 2014). Our study revealed a downregulation of renal Sirt1 expression in the Akita mice which is in accordance with the earlier studies (Chuang et al., 2011; Hasegawa et al., 2013; Yacoub et al., 2014). Interestingly, Nimbidiol normalized Sirt1 expression in Akita mice, suggesting its potential renoprotective role in diabetes.

Further, our present study showed a distinct upregulation in the renal expression of biomarkers related to kidney-damage viz. KIM-1 and LCN-2 in the Akita mice, which is in agreement with the previous studies (Nielsen et al., 2010; Liu et al., 2022). Interestingly, Nimbidiol mitigated KIM-1 and LCN-2 levels in the kidney of Akita mice, indicating the therapeutic potential of Nimbidiol to ameliorate diabetic renal injury.

A plethora of studies have indicated the concomitant contribution of oxidative stress and inflammation in the pathogenesis of diabetes and hypertension (Vaziri and Rodriguez-Iturbe, 2006; Kern, 2007). It is well established that conjunction of diabetes and hypertension induces oxidative stress and inflammation, that synergistically leads to pathogenesis of DN. The close relationship between oxidative stress and inflammation has been indicated by many studies (Vaziri and Rodriguez-Iturbe, 2006; Kern, 2007). The production of ROS by inflammatory molecules has been suggested to trigger the oxidative stress, that in turn, induces the NF-κB-mediated upregulation of pro-inflammatory cytokines and chemokines (Vaziri and Rodriguez-Iturbe, 2006; Kern, 2007). In agreement with these previous reports, our study also showed that an elevated ROS generation was accompanied by an upregulation of p-NF-κB, downregulation of IkBα, along with an increased production of pro-inflammatory cytokine, IL-17, and a reduction of anti-inflammatory cytokine, IL-10 in the kidney of diabetic mice. Nimbidiol-treatment ameliorated these renal pathophysiological conditions in Akita mice.

**FIGURE 11:**
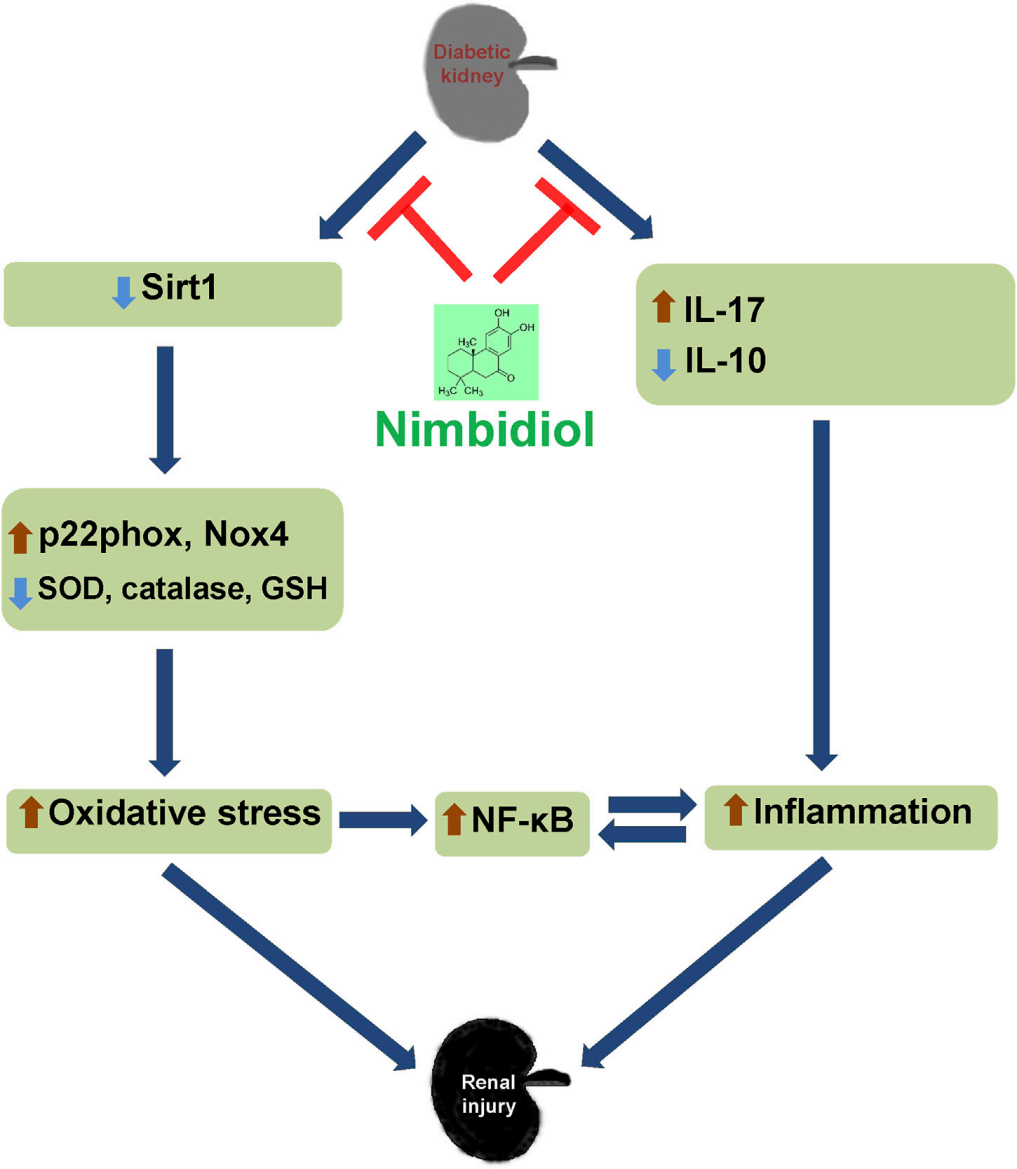
Schematic of the overall findings. Diabetic conditions induce redox imbalance leading to the renal injury. Nimbidiol mitigates redox imbalance and protects kidney by inhibiting NF-κB signaling pathway in type-1 diabetes.

Together, the findings of our present study indicate that diabetic conditions facilitate to the elevated blood pressure, increased renal resistance, and decreased renal vasculature in Akita mice. Further, 4HNE, p22phox, Nox4, and ROMO1 were increased in diabetic mice, which was associated with decreased GSH: GSSG, SOD, catalase, eNOS, Sirt1, ACE2, and increased iNOS levels and ultimately leading to the oxidative stress and inflammation mediated renal damage as evidenced by the elevated kidney injury markers such as LCN-2 and KIM-1. Nimbidiol ameliorated these pathological changes. The present study thus indicates that oxidative stress plays a crucial role to promote diabetic renal injury, and Nimbidiol alleviates redox imbalance and thereby protects kidney in part by inhibiting NF-κB signaling pathway in T1D (Figure 11). Therefore, the novel glucosidase inhibitor, Nimbidiol could be used as a potential anti-oxidative agent for the treatment of diabetic kidney injury in future.

## Data Availability

The original contributions presented in the study are included in the article/Supplementary Material, further inquiries can be directed to the corresponding author.
